# Supplementary material for: l-Carnitine and Acetyl-l-Carnitine Induce Metabolism Alteration and Mitophagy-Related Cell Death in Colorectal Cancer Cells
Source: Nutrients. 2025 Mar 13;17(6):1010. doi: 10.3390/nu17061010 (PMC11946136; doi:10.3390/nu17061010)
Supplement: Supplementary file 1 [file nutrients-17-01010-s001.zip › nutrients-3511346-supplementary.docx]

l-Carnitine and Acetyl-l-Carnitine Induce Metabolism
Alteration and Mitophagy-Related Cell Death
in Colorectal Cancer Cells

Isabella Donisi ^1^, Anna Balestrieri ^2^, Vitale Del Vecchio ^3^, Giovanna Bifulco ^4^, Maria Luisa Balestrieri ^1,^*,
Giuseppe Campanile ^4^ and Nunzia D’Onofrio ^1^

^1^ Department of Precision Medicine, University of Campania Luigi Vanvitelli, Via Luigi De Crecchio 7, 80138 Naples, Italy; isabella.donisi@unicampania.it (I.D.); nunzia.donofrio@unicampania.it (N.D.)

^2^ Food Safety Department, Istituto Zooprofilattico Sperimentale del Mezzogiorno,
80055 Portici, Italy; anna.balestrieri@izsmportici.it

^3^ Department of Experimental Medicine, University of Campania Luigi Vanvitelli, Via Luciano Armanni 5, 80138 Naples, Italy; vitale.delvecchio@unicampania.it

^4^ Department of Veterinary Medicine and Animal Production, University of Naples Federico II,
80137 Naples, Italy; giovanna.bifulco@unina.it (G.B.); giuseppe.campanile@unina.it (G.C.)

***** Correspondence: marialuisa.balestrieri@unicampania.it

**Supplementary Materials**


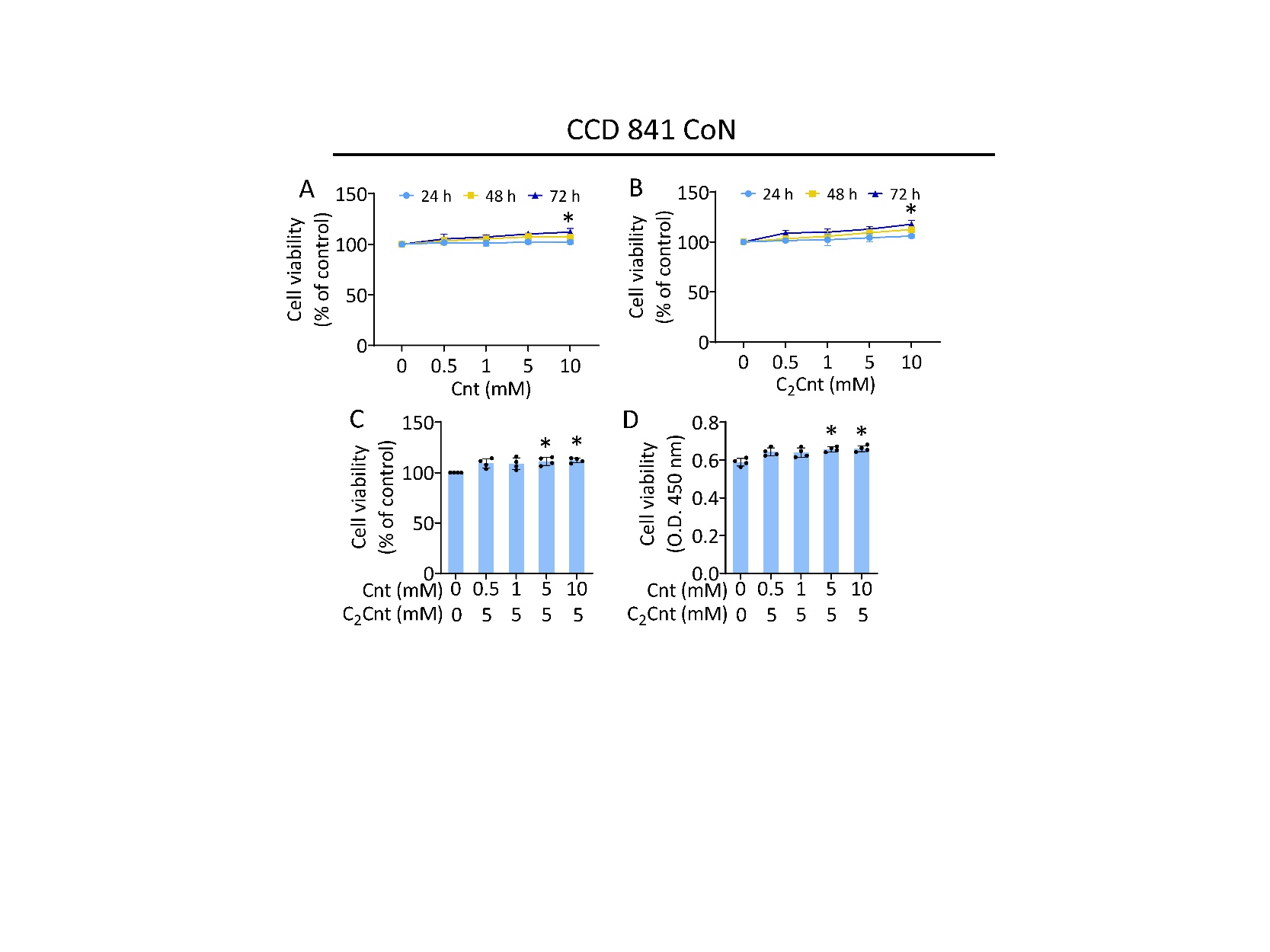


**Figure S1.** Effect of carnitines on colon cell viability. (A, B) Cell viability assays performed on normal colon epithelial cells (CCD 841 CoN) after exposure to different concentrations of Cnt or C_2_Cnt (up to 10 mM) for different times (24, 48 and 72 h). (C, D) Cell viability of CCD 841 CoN after treatments with Cnt and C_2_Cnt in combination. Control cells were grown in medium containing the same volume of HBSS-10 mM Hepes. The data are expressed as the mean ± SD of *n* = 4 independent experiments. *p < 0.05, indicating significant differences between 0 μg/mL (or Control) and sample treatments (Cnt/C_2_Cnt).


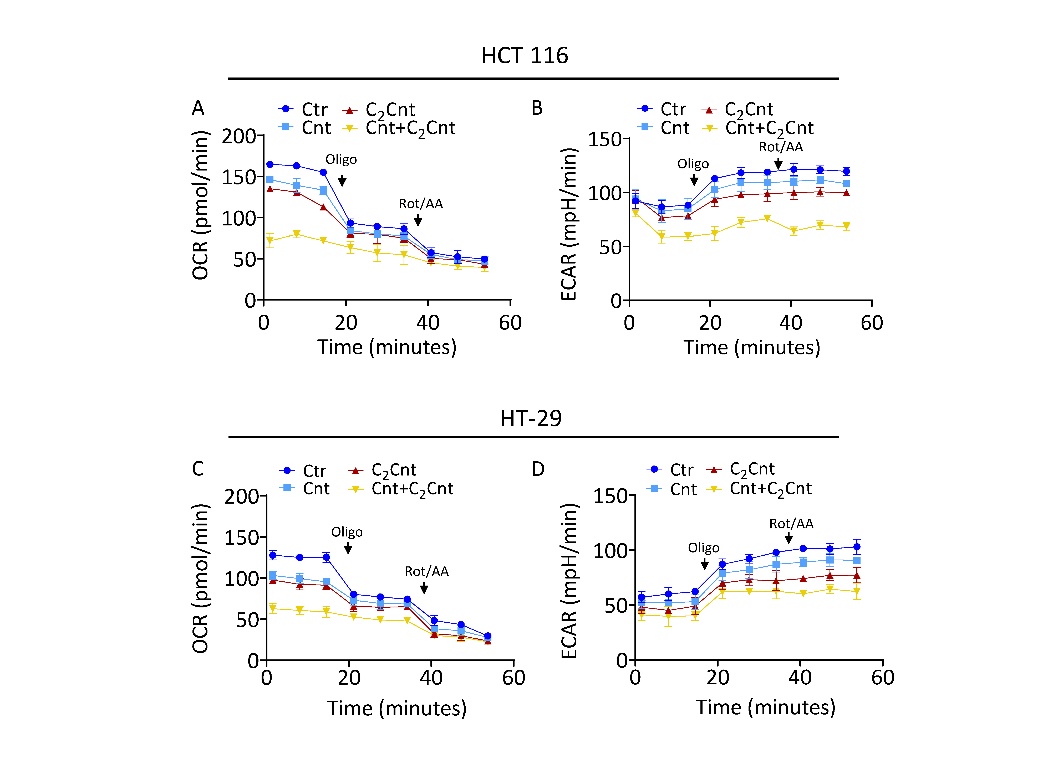


**Figure S2.** Carnitines effects on mitochondrial metabolism state. (A,C) Oxygen consumption rate (OCR) and (B,D) extracellular acetylation rate (ECAR) assessed with Seahorse analyzer in HCT 116 and HT-29 cells treated with Cnt and C_2_Cnt alone or in combination.


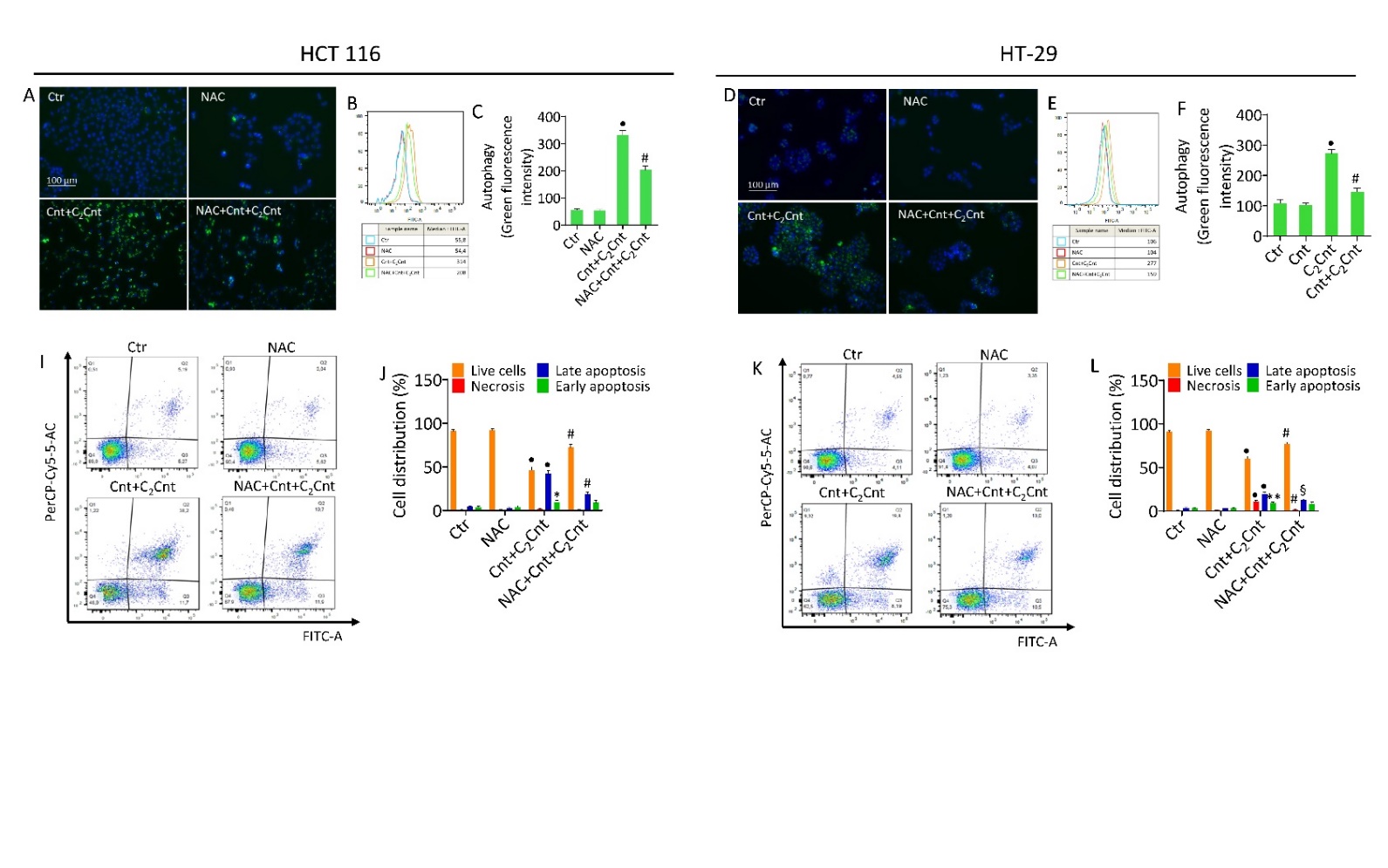


**Figure S3.** Suppression of oxidative stress ameliorated cell death. Representative fluorescent images and FACS analysis of autophagy in (A-C) HCT 116 and (D-F) HT-29 cell lines pre-treated with NAC and then with Cnt+C_2_Cnt for 72 h. (I-L) Dot plots and relative histograms of annexin V-FITC and PI-staining. Data are expressed as mean ± SD of n = 3 experiments. Q1: necrotic cells; Q2: late apoptotic cells; Q3: early apoptotic cells; Q4: viable cells. Scale bars = 100 μm. • p < 0.001, indicating significant differences between Control (Ctr) and sample treatments (Cnt/C_2_Cnt); § p < 0.05, indicating significant differences between NAC+Cnt+C_2_Cnt and combined treatments (Cnt+C_2_Cnt); # p < 0.01 indicating significant differences between NAC+Cnt+C_2_Cnt and combined treatments (Cnt+C_2_Cnt).
